# Adhesive and Rheological Features of Ecofriendly Coatings with Antifouling Properties

**DOI:** 10.3390/polym15112456

**Published:** 2023-05-25

**Authors:** Cristina Scolaro, Leonarda Francesca Liotta, Carla Calabrese, Giuseppe Marcì, Annamaria Visco

**Affiliations:** 1 Department of Engineering, University of Messina, Contrada Di Dio, 98166 Messina, Italy; 2 Istituto per lo Studio dei Materiali Nanostrutturati (ISMN)-CNR, Via Ugo La Malfa 153, 90146 Palermo, Italy; leonardafrancesca.liotta@cnr.it (L.F.L.); carla.calabrese@ismn.cnr.it (C.C.); 3 “Schiavello-Grillone” Photocatalysis Group, Department of Engineering, University of Palermo, Viale Delle Scienze, 90128 Palermo, Italy; giuseppe.marci@unipa.it; 4 Institute for Polymers, Composites and Biomaterials, CNR-IPCB, Via P. Gaifami 18, 9-95126 Catania, Italy

**Keywords:** antifouling, ecofriendly, coating, pull-off, cross-cut test, rheology

## Abstract

In this work, formulations of “environmentally compatible” silicone-based antifouling, synthesized in the laboratory and based on copper and silver on silica/titania oxides, have been characterized. These formulations are capable of replacing the non-ecological antifouling paints currently available on the market. The texture properties and the morphological analysis of these powders with an antifouling action indicate that their activity is linked to the nanometric size of the particles and to the homogeneous dispersion of the metal on the substrate. The presence of two metal species on the same support limits the formation of nanometric species and, therefore, the formation of homogeneous compounds. The presence of the antifouling filler, specifically the one based on titania (TiO_2_) and silver (Ag), facilitates the achievement of a higher degree of cross-linking of the resin, and therefore, a better compactness and completeness of the coating than that attained with the pure resin. Thus, a high degree of adhesion to the tie-coat and, consequently, to the steel support used for the construction of the boats was achieved in the presence of the silver–titania antifouling.

## 1. Introduction

The term biofouling refers to the undesirable phenomenon that leads to the growth of marine micro-organisms on all surfaces immersed in the sea, from boats to oil platforms, to submarine pipes, etc. [1,2,3]. The biofouling phenomenon of surfaces immersed in seawater begins with the adhesion of small bacterial species (microfouling) which grow, forming a biofilm caused by the multiplication of bacterial cells and by the synthesis of extracellular polymeric substances, thanks to the presence of specific soluble organic substances in an aqueous environment (macrofouling) [4]. The progressive depositing of marine micro-organisms creates an economic and environmental problem if specific antifouling paints are used which contain biocides that are not environmentally friendly and, therefore, harmful to marine ecosystems [5,6]. Other problems for the marine environment are related to the higher fuel consumption in boats with higher marine organism adhesion to maintain their speed of movement compared to the boats without fouling [7].

The biocides available on the market are very effective in hindering the proliferation of marine organisms, but at the same time, they also indirectly destroy all the other marine organisms, with serious environmental damage [8,9]. The coatings with biocides used on the “underwater” parts in the naval industry are typically based on silicone, polyurethane, epoxy resin, etc. [10,11,12,13]. These coatings, whether with antifouling action (AF) or fouling release action (FR), must adhere perfectly to the metal support of the boats, must resist over time, and must be capable of eliminating the problem of the encrusting organisms [14]. In 2011, the International Maritime Organization (IMO) published the resolution MEPC.207 (62) that states Biofouling Guidelines to control and manage biofouling on ships [15]. Legislation has banned highly effective antifouling paints based on metallic biocides, such as tributyltin and other organotin compounds, with the intention of a “greener” alternative biocide. However, Russell G. Uc-Peraza et al. highlighted in 2022 that despite the efforts of the IMO and the Rotterdam Convention to ban the use and trade of tributyltin (TBT)-based antifouling paints, the situation persists and seems to be getting worse [16]. In a recent study, researchers identified 25 active ingredients currently used as biocides, with some formulations containing up to six biocides being used together. The most common ones were cuprous oxide, copper pyrithione, zinc pyrithione, zineb, DCOIT, and cuprous thiocyanate. They reported that cuprous oxide was the most frequent biocide, with a mean relative concentration of 35.9 ± 12.8% (*w*/*w*) in antifouling paint formulations. As a result, researchers have been looking for alternative paints that use a low content of metallic biocides, non-toxic inorganic oxides or quaternary ammonium salts as active additives. 

Several studies have been published in the literature with the aim of producing ecofriendly coatings with chemical green features and excellent physical–mechanical performance [16,17,18,19,20,21,22]. Among the permissible materials, nanocomposites based on Cu/TiO_2_ and Ag/TiO_2_ oxides with a low metal content (≤5 wt%) have been studied for their antifouling properties. These nanocomposites exhibit remarkable properties that resist the adhesion and growth of marine organisms, such as algae, barnacles, and mussels, which can affect the performance of marine structures, ships, and underwater pipelines [23,24,25,26,27,28]. Recently, our research group highlighted the antifouling and antimicrobial activity of Ag, Cu and Fe nanoparticles supported on silica and titania [29]. We reported that the selection of silica and titania as carriers of active nanoparticles is based on their adjustable surface properties, such as high specific surface area and hydrophobic/hydrophilic balance, in addition to their intrinsic parameters like high thermal stability, chemical stability, and biological stability. Then, we deposited Cu and Ag nanoparticles over commercial silica, titania, and mixed silica–titania powders and investigated their antifouling properties and preliminary rheological features [23]. The titania-based coatings showed better adhesion and workability than silica-based coatings, and the addition of fillers increased the resin viscosity. The ecotoxicity of the powders was tested using a Microtox luminescence test, excluding the release of toxic substances. The microbiological activity was studied with tests on bacterial growth of various species. The Cu/TiO_2_ powder exhibited the best performance in inhibiting bacteria proliferation. This was attributed to the presence of well-dispersed CuO species in a synergistic interaction with titania.

The present work represents the completion of a scientific investigation that builds upon the previous work of our research group on the same materials with antifouling properties discussed above [23]. In this work, we studied the adhesive and rheological characteristics of nanomaterials based on Cu and Ag as antifouling fillers on commercial silica and titania oxides. Our chemical, physical and mechanical characterization helped us select the best composition of the material among those studied. The optimal filler dispersed in a commercially available thermosetting silicone matrix could represent an innovative environmentally friendly antifouling material that has been tested from several perspectives, including the material’s plan and application, and could potentially replace currently available non-ecofriendly antifouling paints.

## 2. Materials and Methods

### 2.1. Antifouling Filler Preparation and Characterization

The commercial silica was purchased from Merck (Darmstadt, Germany) (amorphous silica gel 60). All the other chemicals used for the synthesis were provided by Sigma Aldrich (St. Louis, MO, USA) with 99.99% purity and were used without any further purification. According to our recently published study [23], silver- and copper-based antifouling powders were prepared using the wetness impregnation method. The bare supports, commercial silica and titania oxide were impregnated with the proper amount of aqueous solutions of Cu(NO_3_)_2_·2.5H_2_O and AgNO_3_ precursors, in order to obtain the desired copper and silver loadings, corresponding to Cu 5 wt% and/or Cu and Ag 2.5 wt%. The resulting materials were dried at 120 °C overnight and finally calcined at 500 °C for 2 h with a heating ramp of 5 °C/min. According to the labels previously used, the materials were named SMx (x = 1, 5, 6, 7); their chemical composition in terms of Cu and Ag weight % (wt%) and atomic % (at%) is listed in Table 1. 

Specific surface area (SSA), pore volume and mean pore diameter of the materials were determined with N_2_ physisorption analysis at −196 °C using the ASAP 2020 equipment (Micromeritics, Norcross, GA, USA) (Micromeritics, United States). Prior to the analysis, the samples were outgassed at 200 °C under vacuum for 2 h. The specific surface area (SSA) was calculated via the Brunauer–Emmett–Teller (BET) method in the standard pressure range of 0.05–0.3 P/P_0_. The pore volume and pore size distribution (pore width) were obtained through analysis of the desorption branch, using the Barrett, Joyner and Halenda (BJH) calculation method. 

Scanning electron microscopy (SEM) was performed by using a FEI Quanta 200 ESEM microscope, operating at 20 kV on specimens upon which a thin layer of gold had been evaporated. An electron microprobe used in an energy dispersive mode (EDX) was employed to detect and quantify the actual content of silver and copper present in the various antifouling fillers prepared.

### 2.2. Antifouling Coating Preparation

The SMx (x = 1, 5, 6, 7) synthetic antifouling fillers (0.1 wt%) were individually mechanically dispersed within a commercial resin based on silicone and biocide-free (Hempel’s Silic One, HEMPEL S.R.L Genova, Italy top-coat, blu-color) [30]. 

**Figure 1:**
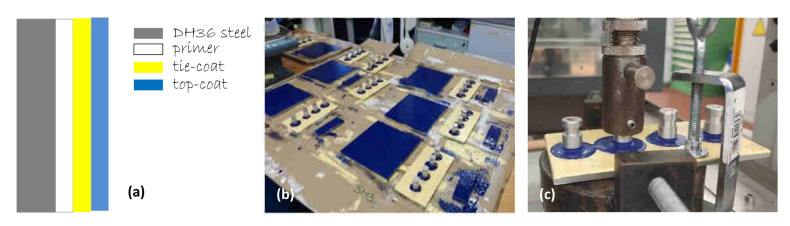
Scheme of layers deposited on the DH36 steel (**a**); image of the painted steel samples for the mechanical adhesion tests (**b**); image of the experimental set-up for the pull-off test (**c**).

A shipbuilding steel plate (DH34 steel, 150 mm × 75 mm, 5 mm thickness) usually used in offshore and marine constructions, supplied by Fincantieri S.p.a., was pre-treated with a white color layer of primer (Hempel’s Light Primer, HEMPEL S.R.L Genova, Italy, ~116 μm thick) and a second yellow color layer of tie-coat (Hempel’s Silic One, HEMPEL S.R.L Genova, Italy, ~180 μm thick), before the deposition of the third blue topcoat layer with the various synthetic antifouling fillers (see Figure 1a).

### 2.3. Coatings Characterization

A digital thickness gauge (SAMA Tools-SA8850, SAMA Italia, Viareggio, Italy) was used for measuring the thickness of antifouling coatings on rectangular steel specimen. A map was drawn on the specimen, covered with the antifouling coating, which identifies a grid of 84 (14 × 6) points. Thickness measurements were made by placing the probe perpendicular to the specimen at each point of the resulting grid and afterwards calculating the mean values of all measurements.

The optical microscope used for the observation and study of the morphology of the coatings in question is the Hirox digital microscope mod. KH8700 (Hirox, Tokyo, Japan) mounting a 103 MX(G)-5040Z lens at room temperature. It is equipped with special optics and with the possibility of mapping and measuring xyz (3D).

A rotational rheometer (MC-502, Anton Paar, Graz, Austria) was utilized to analyze the flow behavior of antifouling coatings at different temperatures. Measurements for this work were carried out by using the plate–plate geometry. Static viscosity tests with varying temperatures (−25 °C/+ 100 °C) were carried out with this rheometer. Each viscosity test was performed at an ambient temperature of 25 °C within the shear rate of 0.1 s^−1^ to 1000 s^−1^. The Amplitude Sweep Stability test, as a function of deformation (stress), was conducted at 1 Hz from 1 Pa to 100,000 Pa of stress. This test allowed us to calculate LVR (Linear Viscoelastic Region). The Temperature Sweep Test (frequency of 1 Hz) was performed by varying the temperature from 25° to 100 °C, in an initial time interval of 1 min and final time of 10 min, at 1.3 Pa (linear viscoelastic region (LVR) obtained in the previous test).

The cross-cut test has been performed to evaluate the adhesion of coating films to a metallic DH36 steel substrate by using a commercial Cross Hatch Adhesion Tester (SAMA Tools SADT502-5, SAMA Italia, Viareggio, Italy) according to ASTM D3359e2 “Standard Test Method for Measuring Adhesion by Tape Test”. A grid incision was made (horizontally and vertically spaced 2 mm in both cases) in a test area of approximately 10 × 10 cm. Subsequently, a 3M adhesive tape was stuck onto the cutting grid and removed with an even peeling movement. The test is evaluated by comparing the sectional grid image with the reference images from ISO 2409:2013. Depending on the condition of the damage, a cross-cut parameter from 0 (very good adhesive strength) to 5 (very poor adhesive strength) is assigned according to the number of squares that have flaked off and the appearance. 

The pull-off test has been performed with a LLOYD LR10K Universal Dynamometer machine (Ametek-Lloyd Instruments Ltd., Fareham Hampshire, UK) with a load cell of 10 KN, pre-load of 1.00 N, and speed of 1 mm/min. A steel metal dolly was attached perpendicularly to a DH36 steel metal sheet (80 × 10 mm, thickness 5 mm) in accordance with ASTM D4541-02 (or ISO 4624:2016). Mechanical values are the result of the average calculated on 6 specimens for each type of topcoat analyzed.

Prism 8.0.2 statistical software (GraphPad, Inc, La Jolla, CA, USA) was used for the statistical analysis. Data are reported as mean ± SD (±Standard Deviation) at a significance level of *p* < 0.05. The D’Agostino and Pearson test was used for normality test of data, and the Brown–Forsythe test for homogeneity of the variance test. Since all data used in this study satisfied these two tests, the one-way analysis of variance (ANOVA) with Bonferroni’s post hoc test was performed to evaluate the statistical significance of the differences between the groups (significance level: 0.05).

## 3. Results

### 3.1. Evaluation of the Morphological Properties of the SM Powders

**Figure 2:**
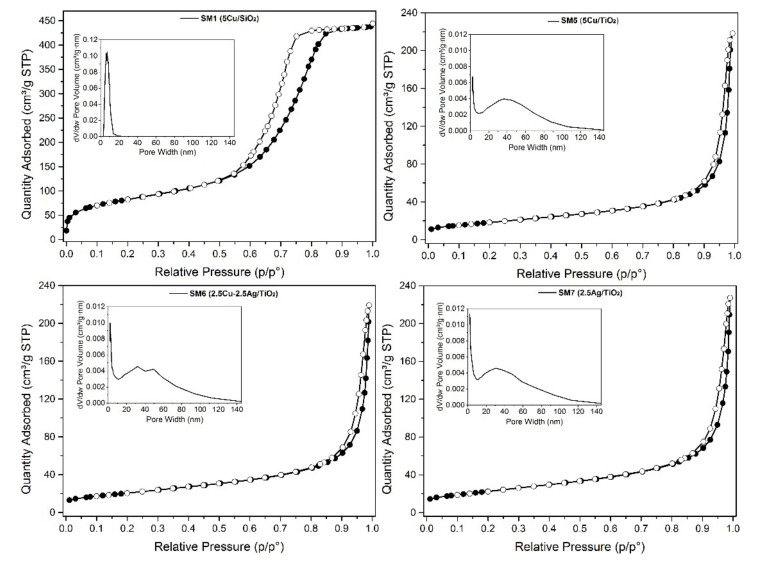
N_2_-adsorption/desorption isotherms of SM1, SM5, SM6 and SM7. Pore size distribution (inset).

As previously reported [23], the so-prepared samples were characterized by X-ray diffraction (XRD), X-ray photoelectron spectroscopy (XPS) and temperature programmed reduction (TPR). The results confirm the data already published, so no further details are herein reported. As it concerns the textural properties, we have considered it worthy of investigation to go deeper in the discussion of the results with respect to the data listed in ref. [23], considering that the morphology can influence the rheological and adhesive properties of the resulting coatings. In Table 2, the SSA, pore volume and pore width values are listed; in Figure 2, the adsorption/desorption profiles and pore size distribution (in the inset) are shown. The isotherms are type IV, according to IUPAC classification, with hysteresis typical of mesoporous materials, especially for the silica-based one (SM1).

According to the specific surface area of bare silica (320 m^2^g^−1^), Cu/SiO_2_ (SM1) displays high specific surface area, equal to 300 m^2^g^−1^, with pore volume of 0.68 cm^3^g^−1^, and a relatively narrow pore size distribution centered at 6.9 nm. The titania-based materials, Cu, Ag, and Cu-Ag/TiO_2_ (SM5–7), exhibited slightly lower specific surface areas than bare TiO_2_ (45–48 versus 56 m^2^g^−1^) and pore volume values around 0.47 cm^3^g^−1^ [23]. 

**Figure 3:**
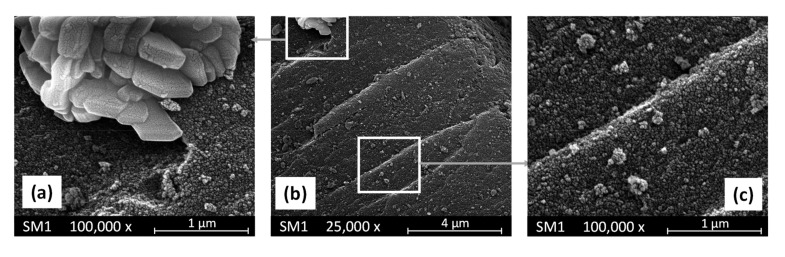
SEM pictures of SM1 sample at two different magnifications. Pictures (**a**,**c**) report enlargements of the two areas evidenced in picture (**b**).

The morphology of the antifouling fillers was studied through SEM pictures. Figure 3 reports some SEM pictures of the SM1 sample. This material consists of large agglomerates (sizes between 40–100 mm) of nanoparticles (20–40 nm) of SiO_2_ on the surface of which there are islands of agglomerates of large crystals (see Figure 3a) rich in copper as evidenced by the EDX investigation. These crystals probably constitute of copper oxide. Consequently, the composition of this material is very inhomogeneous, although the average content of Cu measured by the EDX resulted only slightly higher than the nominal value listed in Table 1.

**Figure 4:**
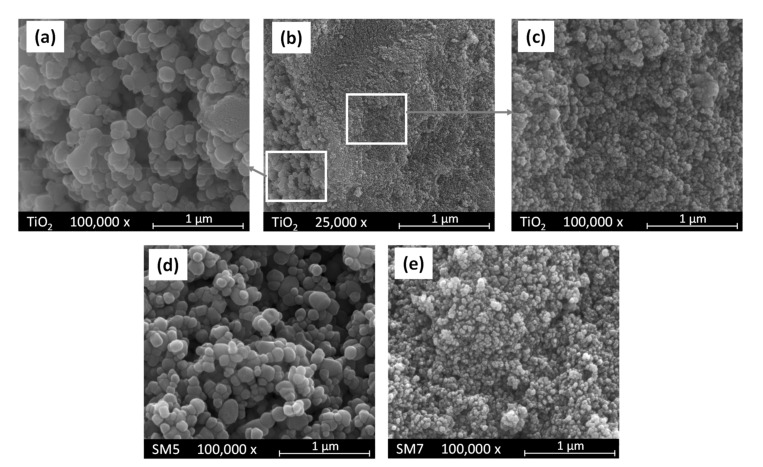
SEM pictures of (**a**–**c**) bare TiO_2_ at two different magnifications, (**d**) SM5 and (**e**) SM7 samples. Pictures (**a**,**c**) report enlargements of the two areas evidenced in picture (**b**).

In panels (a), (b) and (c) of Figure 4, three pictures are reported of the bare TiO_2_ sample used as support of the SM5 and SM7 materials, whose pictures are reported in the same Figure 4 in panels (d) and (e), respectively. As it is possible to observe, the bare TiO_2_ sample constitutes of agglomerates of nanoparticles with different sizes from ca. 20 to ca. 200 nm (see panels (a) and (c)). Interestingly, the SM5 and SM7 samples obtained by loading TiO_2_ with Cu and Ag, respectively, show the same morphology of bare TiO_2_ as evidenced by the pictures reported in panels (d) and (e) of Figure 4.

The EDX analysis indicates that, only in the case of the SM7 sample, silver is uniformly deposited onto the surface of TiO_2,_ and its amount is practically equal to the nominal one. On the other hand, the distribution of copper onto the TiO_2_ surface in the SM5 sample appears not uniform, resulting, moreover, in the average content of copper slightly higher with respect to the nominal value reported in Table 1. As far as the SM6 sample is concerned, the SEM pictures, not reported for the sake of brevity, indicate that the morphology of this material is also in this case very similar to that of the bare TiO_2_ support, but the distribution of Cu and Ag onto the TiO_2_ surface is not uniform although their average content is very close to the nominal one (see Table 1).

### 3.2. Evaluation of the Adhesion Power and Rheological Features of the Coatings

The evaluation of the adhesive properties of the coatings was carried out through two types of mechanical cross-cut and pull-off tests. As we are going to see, both tests showed that the coatings we made in the laboratory (by mixing the antifouling fillers with the commercial silicone resin, H) gave an improvement in terms of adhesive properties. 

**Figure 5:**
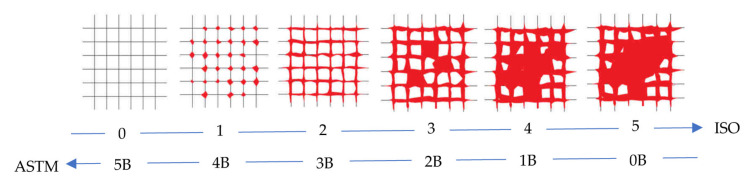
Cross-cut test levels according to ISO and ASTM scales that grows in the arrow’s direction.

With regard to the cross-cut test, the adhesion level is classified according to the ASTM and ISO standards already discussed in Section 2.3 of Materials and Methods, with the scale shown in Figure 5 [31,32].

**Figure 6:**
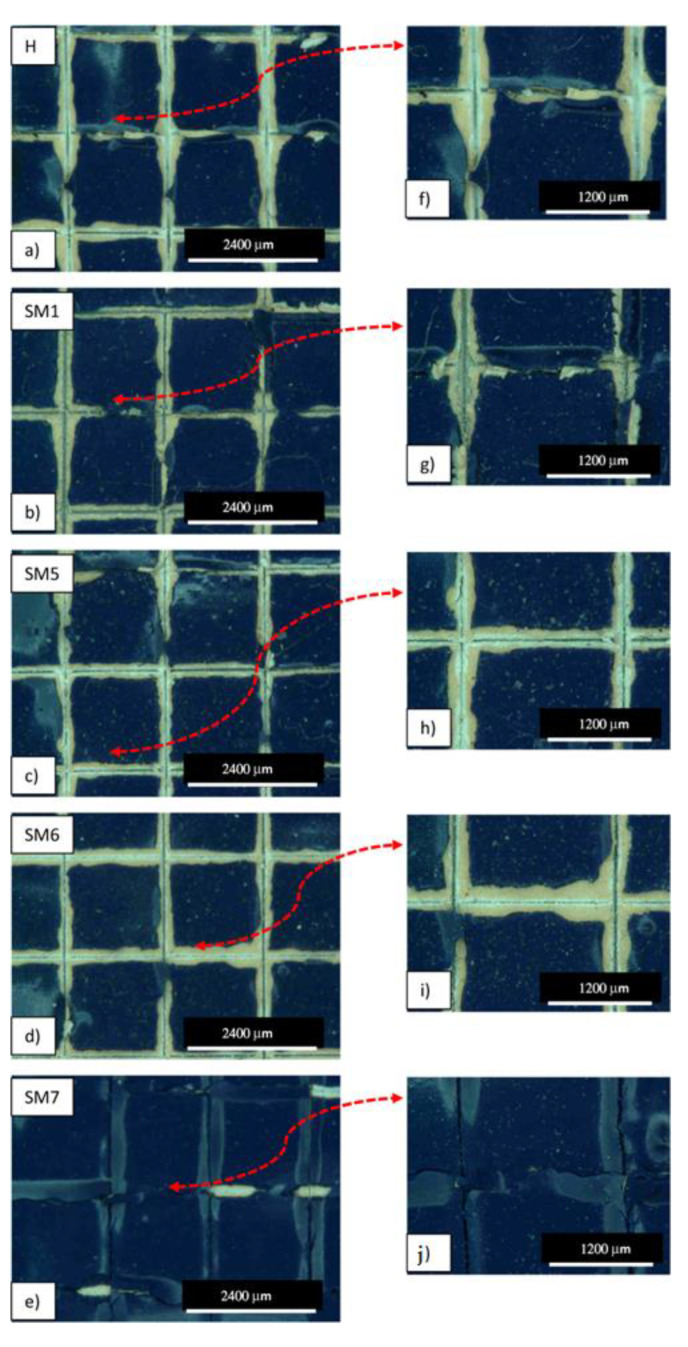
Optical microscope images of the adhesion cross-cut test of commercial topcoats H, HSM1, HSM5, HSM6 and HSM7, at 50× magnification ((**a**–**e**), respectively), and at 100× magnification ((**f**–**j**), respectively). The red dotted line indicates the corresponding area viewed at higher magnification.

The images of Figure 5 help us to visualize the evaluation of the adhesion level and compare it with the optical images of Figure 6, where the edges of the cuts of all the coating samples are shown at two different magnifications (50× and 100×). After the incision of the cutting edge, large lateral portions around the points of passage of the blade are visibly detached in the commercial sample H (Figure 6a,f). Similarly to sample H, in sample HSM 1 (Figure 6b,g) and in sample HSM6 (Figure 6d,i), large portions of paint are detached along the edges with the involvement of larger areas. In HSM5 (Figure 6c,h), the detaching along the edges involves only the parts of the coating close to the edges of the notch without involving other areas. Although from a visual point of view the difference in adhesion between the sample HSM 5 and the others before discussed (H, HSM1, HSM6) is detectable, this difference is not appreciable with reference to the images of the scale in Figure 5. Therefore, in accordance with the reference standards, the H, HSM1, HSM5, and HSM6 samples fall within a single value of adhesion level that is evaluated as 3B (specifically for the ASTM D 3359-09e2) and ISO-2 (specifically for the ISO 2409:2007) [30]. We can define level 3B/2 as “sufficient” adhesion level, considering the ASTM/ISO scale from 1B/5 to 5B/0 in progressive terms of very poor, poor, sufficient, good, and very good adhesion [31].

The HSM7 specimen appears to exhibit the best level of adhesion among those analyzed because it presents sharper cut areas, with more limited edge-detachment portions than the other coatings discussed above (Figure 6e,l), with a good level of adhesion 4B/1.

To evaluate the level of adhesion of our coatings from a quantitative point of view, a more accurate evaluation was required through the mechanical pull-off test discussed below.

**Figure 7:**
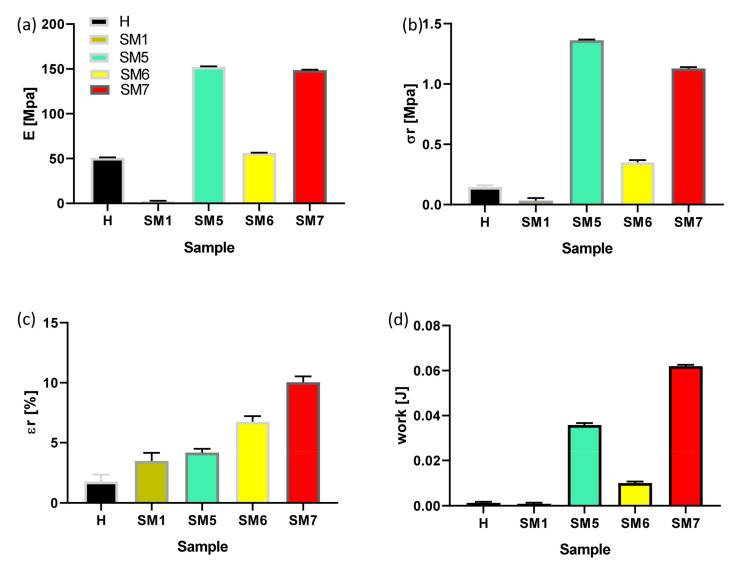
Mechanical parameters of pull-off test of commercial tie-coat H and of HSM(x) topcoats: Young’s modulus E (**a**); the stress at break, σ_r_ (**b**); the deformation at break, ε_r_ (**c**); and the work at break, W_r_ (**d**).

The pull-off test result is detailed in Figure 7. We can see that the coatings based on titania (HSM5, HSM6, HSM7) exhibit better adhesion power than pure resin, H (*p* < 0.0001). In detail, the stiffness of pure resin is ~50 MPa, its tensile strength is ~0.15 MPa, the elongation at break is ~1.8% and the work at break is 1.3 × 10^−3^ J. The HSM7 coating obtained the best values, especially in terms of deformability (ε_r_) and toughness (W_r_), with a high value of strength (σ_r_) among all those analyzed (*p* < 0.0001), according to cross-cut test results. The HSM1 coating, which contains silica, on the other hand, gave the worst results in terms of adhesion. Its stiffness, mechanical strength and ductility are inferior to all coatings, including the commercial silicone one.

The higher adhesion power of SM7 compared to the other coatings (checked by both the adhesion tests discussed above) is the reason why our attention was focused on the HSM7 sample only, whose mechanical strength and modulus are ~1.3 MPa and ~150 MPa, respectively (*p* < 0.0001). 

The pull-off strength of our ecofriendly coating HSM7 is close to that of silane-based coatings with anticorrosive properties prepared by Arabpour et al., that is 1.25 ± 0.07 MPa [2,33].

Since TiO_2_ is present in SM7, the subsequent in-depth rheological analyses were carried out in pure resin (H), in H + TiO_2_ (or HTiO_2_), and in H + TiO_2_ + Ag (HSM7). The aim was to understand if their adhesive power is linked to the presence of titania or to the presence of the metal deposited on titania (as in HSM7 sample). The rheology is the best choice to detect the structural changes of the polymers because it is very sensitive, even in the presence of modified polymeric structures. Any change, from chain scission to cross-linking, affects the mobility of the macromolecular chains with obvious repercussions on the rheological behavior of the polymer [34,35].

**Figure 8:**
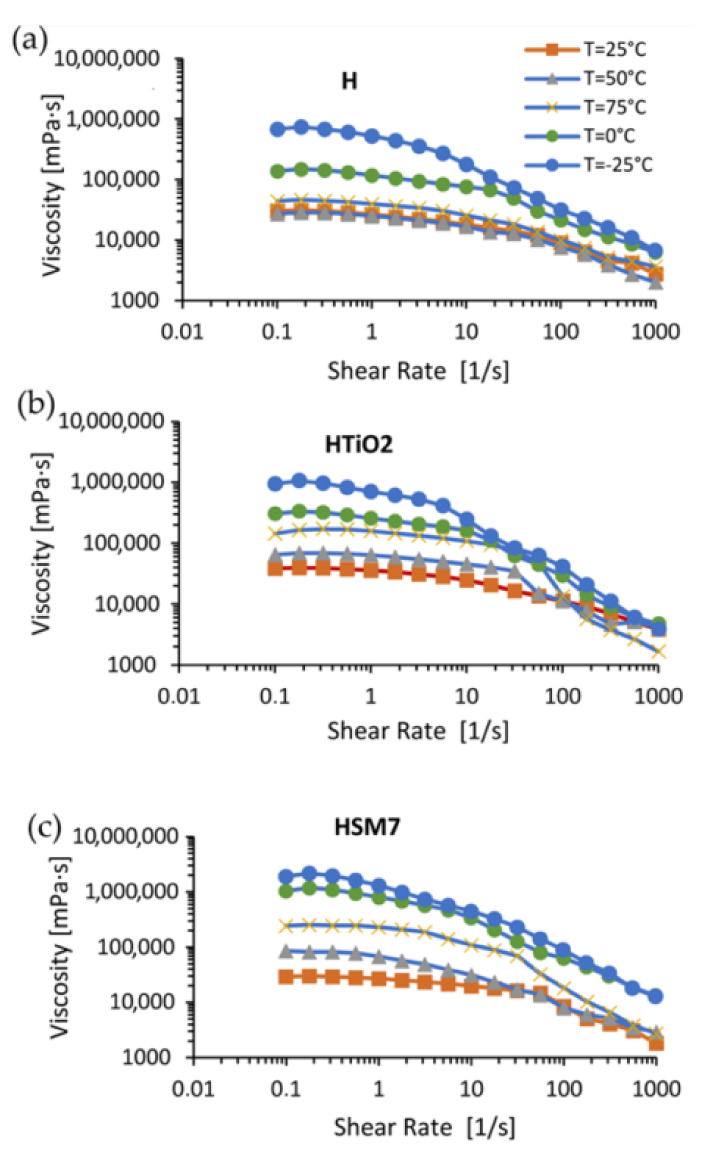
Flow curves (left column) and loss/conservative modulus at different temperatures (right column) of H (**a**), HTiO_2_ (**b**) and HSM7 (**c**).

In Figure 8, we observe the flow curves as the temperature varies (ranging from −25 to +75 °C). The flow curves relating to pure resin H do not vary much at temperatures in the range of 25–50–75 °C, remaining below 40,000 MPa·s. Instead, the temperature lowering to 0 °C and to −25 °C causes an increase in viscosity and a decrease in the extent of the curve at constant viscosity due to the ideal or Newtonian behavior, as expected.

The addition of titania, and of titania with metal (H + TiO_2_ and HMS7 samples, respectively), changes the rheological behavior of the coatings. The viscosity grows even more in the order of H < H + TiO_2_ < H + TiO_2_ + Ag. The filler presence could act like a catalyzer of the chemical resin’s cross-linking reaction to explain the viscosity grown. This finding agrees with the TiO_2_ ability in generating hydroxyl radicals that, reacting with the silicone resin molecules, catalyzes the cross-links between the resin chains [36]. Moreover, metal species enhancing the photocatalytic activity of titania can enhance the cross-linking process [37]. 

Additionally, filler presence could improve the consistence of the material causing the viscosity grown but considering that the filler load is very little (0.1 wt%), as reported in Section 2.2, this effect seems to be negligible.

In Table 3, the viscosity trend is numerically detailed: the viscosity of the coatings grows when decreasing the temperature and vice versa, according to the Williams–Landel–Ferry or WLF equation [38]. This effect is quantitatively similar in all the coatings: the viscosity of pure H resin at −25 °C (6.86 · 10^5^ mPa·s) decreases up to 0.44 · 10^5^ mPa·s at +75 °C (93.6%). Similarly, the viscosity of HMS7 at −25 °C (19.1 · 10^5^ mPa·s) decreases up to 2.44 · 10^5^ mPa·s at +75 °C (−87.2%).

The addition of TiO_2_ and, even more, the addition of TiO_2_ + Ag improves the coating’s viscosity. At the lowest temperature of −25 °C, the viscosity of pure H coating grows from the value of 6.86 · 10^5^ mPa·s, up to 19.1 · 10^5^ mPa·s in H + TiO_2_ + Ag (+178%). At the highest temperature of +75 °C, the viscosity of pure H coating improves from the value of 0.44 · 10^5^ mPa·s, up to 2.44 · 10^5^ mPa·s in H + TiO_2_ + Ag (+454%). This notable raise in viscosity, despite the temperature being the highest of all those studied, i.e., 75 degrees centigrade, is justifiable considering the hypothesis that the presence of the filler can facilitate the cross-linking of the resin. It is in fact known that as the degree of cross-linking increases, the structural complexity of the macromolecule rises and therefore, the difficulty of sliding of the coating increases, represented by a higher viscosity [38]. 

The properties of the material are linked to its intrinsic structure. Therefore, the higher the compactness and the degree of cross-linking of our coatings (and the higher its resulting viscosity), the higher the level of adhesion to the tie-coat overlaying shipbuilding stainless steel.

**Figure 9:**
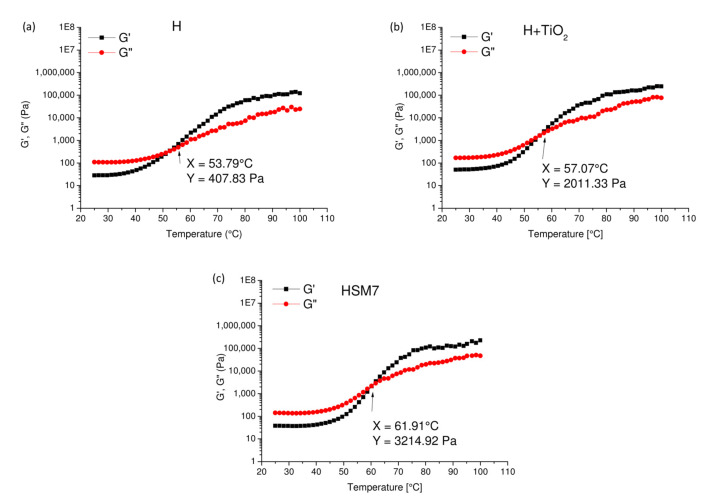
Conservative (G′) and loss modulus (G″) at different temperatures of pure resin, H (**a**), H + TiO_2_ (**b**) and of H + TiO_2_ + Ag (HSM7) (**c**).

Finally, in Figure 9 we observe the conservative modulus (G′) and the viscosity modulus (G″) as a function of temperature. The cross-over point occurs again in the order:

H < H + TiO_2_ < H + TiO_2_ + Ag,

at 53.79 °C and 408 Pa (Figure 9a), 57.07 °C and 2011 Pa (Figure 9b), and at 61.91 °C and 3215 Pa (Figure 9c). This result agrees with rheological results of Figure 8 and confirms that the titania and, even more, the titania/silver nanoparticles’ presence, improves the consistence/cross-linking degree of the coating since it requires growing conditions of temperature and force to reach the cross-over point [35]. The higher compactness and completeness of the coating, due to a higher cross-linking degree, regulates a consequent better adhesion to the substrate.

## 4. Conclusions

In the present study, we have focused on the application of selected “environmentally friendly” nanomaterials as antifouling fillers based on copper and silver nanoparticles deposited on silica and titania oxides. This research was built on our previous results [23], with the aim to create biocide materials able to replace the currently available non-ecological antifouling paints.

The texture properties of the antifouling formulations were analyzed together with an SEM morphological investigation to understand their effectiveness. The results suggested that the innovative antifouling powders actively resist fouling due to the nanometric size of the particles and the homogeneous dispersion of the metal on the substrate. However, the simultaneous presence of two metal species on the same support limits their dispersion at the nanometric level and the formation of homogeneous compounds. The adhesion mechanical and rheological characterization tests indicated that the adhesion strength to the tie-coat layer improves compared to the pure resin after the addition of TiO_2_ and Ag. Such filler, with antifouling properties, also ameliorates the adhesion of the coating to the tie-coat and the steel support, which are used for boat construction. 

The innovative aspect of this study was the approach to designing the material with the direct application as an antifouling paint. The approach was to analyze the same preparation from a variety of perspectives, including chemical, physical, biological and rheological studies, to understand the interaction between matrix, filler and the final properties of the coating. This approach enabled us to guide the large-scale design of this material.

Remarkably, even low content (0.1 percent by weight) has shown appreciable results, suggesting that small quantities are sufficient to have an appreciable effect in terms of adhesion. Furthermore, this study showed that the best catalyst was the one based on titania with silver, which is certainly less harmful than copper.

Although Cu- and Ag-based oxide materials have shown potential as antifouling agents, there is a need for further research to optimize their properties and to better understand their mechanism of action against marine fouling organisms. Further research could also explore the potential of these oxides as part of a multi-component antifouling system, in combination with other materials and strategies, to optimize the optimal filler’s composition. To achieve this goal, more in-depth research on the long-term biological features of these materials is necessary. Such research could provide valuable information on the efficacy and environmental impact of these materials over extended periods.

## Figures and Tables

**Table 1:** Label and chemical composition (nominal and actual) of the prepared fillers.

		Chemical Composition		
Sample	(Code)	Nominal (wt%)	Nominal (at%)	* Actual (at%)
Cu/SiO_2_	(SM1)	Cu (5.0)	4.7	6.4
Cu/TiO_2_	(SM5)	Cu (5.0)	6.2	10
Cu-Ag/TiO_2_	(SM6)	Cu (2.5)-Ag (2.5)	Cu (3.1)-Ag (1.9)	Cu (3.6)-Ag (1.6)
Ag/TiO_2_	(SM7)	Ag (2.5)	1.9	1.9

* Average values measured with EDX analysis.

**Table 2:** Textural properties of samples from N_2_-adsorption/desorption data.

Sample	(Code)	SSA (BET Method)	Pore Volume (BJH Method)	Pore Width (BJH Method)
		m^2^/g	cm^3^/g	nm
Cu/SiO_2_	(SM1)	300	0.68	6.9
Cu/TiO_2_	(SM5)	45	0.45	35
Cu-Ag/TiO_2_	(SM6)	46	0.47	40
Ag/TiO_2_	(SM7)	47	0.48	35

**Table 3:** Viscosity of the reference resin H at different temperatures before and after the addition of fillers at 1 wt% (TiO_2_ and TiO_2_ + Ag) at shear rate of 1 s^−1^.

Sample	H	H + TiO_2_	H + TiO_2_ + Ag (HSM7)
T (°C)	*η* (mPa·s) × 10^5^		
−25	6.86	9.42	19.1
0	1.38	3.05	10.4
25	0.30	0.38	0.39
50	0.27	0.65	0.86
75	0.44	1.44	2.44

## Data Availability

The data presented in this study are available on request from the corresponding author.
